# Functional Status Assessment of COPD Based on Ability to Perform Daily Living Activities: A Systematic Review of Paper and Pencil Instruments

**DOI:** 10.5539/gjhs.v8n3p210

**Published:** 2015-08-06

**Authors:** Fateme Monjazebi, Asghar Dalvandi, Abbas Ebadi, Hamid Reza Khankeh, Mahdi Rahgozar, Jörg Richter

**Affiliations:** 1 Department of Nursing, University of Welfare and Rehabilitation Sciences, Tehran, Iran; 2 Behavioral Sciences Research Center and Nursing Faculty, Baqiyatallah University of Medical Sciences, Tehran, Iran; 3 Department of Health in Emergency and Disaster, University of Social Welfare and Rehabilitation Sciences, Tehran, Iran; 4 Department of Statistics, University of Welfare and Rehabilitation Sciences, Tehran, Iran; 5 Department of Psychology, University of Hull, Hull, United Kingdom

**Keywords:** activity of daily living, instrument, paper and pencil instruments, chronic obstructive pulmonary disease, systematic review

## Abstract

**Context:**

Activity of daily living (ADL) is an important predictor of mortality in patients with chronic obstructive pulmonary disease (COPD). Increasing ADL is important in patients with COPD and assessment of ADL is one of the best ways to evaluate the status of COPD patients.

**Objectives:**

The objective of this systematic review was to provide an overview of the psychometric properties of paper and pencil instruments measuring ADL in patients with COPD.

**Data Sources:**

English papers published from 1980 to 2014 regarding ADL in patients with COPD were searched in Web of Science, MEDLINE, Google Scholar, Cochrane, PubMed, ProQuest, and CINAHL databases using the following keywords: “COPD”, “ADL”, “activities of daily living”, “daily activities”, “instrument”, “questionnaire”, “paper-and-pencil instruments”, and “measure”. Following the Internet search, manual search was also done to find article references.

**Study Selection:**

A total of 186 articles were found. Of those, 31 met the inclusion criteria. Full texts of articles meeting the inclusion criteria were studied. Consensus-based standards for the selection of health measurement instruments”(COSMIN) were used to assess the quality of the studies.

**Data Extraction:**

Data extraction form based on research aims developed by researchers and psychometric experts, with 17 questions was used.

**Results:**

In these articles, 14 pen and paper instruments were identified for examining ADL in patients with COPD; of which, 4 dealt directly with ADL while 9 assessed other criteria i.e. dyspnea as ADL indicator. The majority of instruments only dealt with two main dimensions of ADL: Basic Activities of Daily Living (BADL) and Instrumental Activities of Daily Living (IADL), and did not consider Advanced Activities of Daily Living (AADL), which is influenced by cultural and motivational factors.

**Conclusion:**

Despite several ADL instruments identified, complete psychometric processes have only been done in a few of them. Selection of the appropriate instrument should focus on the aim of the study and the target construct.

## 1. Introduction

Chronic obstructive pulmonary disease is a major chronic health problem throughout the world (Vestbo, Hurd, & Rodriguez-Roisin, 2012). Functional status impairment is a common finding in COPD patients (R. Garrod, Marshall, Barley, Fredericks, & Hagan, 2007). Functional status is defined as one's ability to perform normal ADLs, to meet basic needs, play usual roles and maintain and improve health (Leidy, 1994). Functional status is a multidimensional concept, which focuses on the capacity to perform ADLs (Skumlien, Hagelund, Bjortuft, & Ryg, 2006).

Chronic obstructive pulmonary disease is typically accompanied by dyspnea (Gullick & Stainton, 2008) and dyspnea is usually associated with decreased functional status and physical ADLs (Peruzza et al., 2003). Decrease in functional status will ultimately lead to sedentary life and compromised health (Kapella, Larson, Covey, & Alex, 2011).

Studies indicate that 78% of patients with COPD have dyspnea even when walking at home, and are faced with difficulties in ADLs (Álvarez-Gutiérrez et al., 2007). Insufficient physical activity is the main cause of disability, severe loss of pulmonary function, early death (Troosters et al., 2010), anxiety and depression in the elderly with COPD (Stuart, Rogers, Balanos, & Wood, 2011). Various studies have demonstrated associations of more physical activity with reduced mortality rate and fewer hospitalizations in patients with COPD (Haggerty, Stockdale-Woolley, & ZuWallack, 1999). The World Health Organization's Global Initiative for COPD (GOLD) report states that increasing physical activities in everyday life is among the important goals of treatment in patients with COPD (Pitta, Troosters, Spruit, Decramer, & Gosselink, 2005).

Most people with COPD experience a decline in functional status, but little is known about the magnitude of decline or factors that contribute to it (Kapella et al., 2011).

One way to assess the functional status is to ask patients via an ADL questionnaire (Skumlien et al., 2006). Measuring ADL is one of the best ways to evaluate the level of health (Resnick, 2000), assess the progress of the disease, and assess the efficacy of rehabilitation or other treatments in patients with COPD (Janaudis-Ferreira, Beauchamp, Robles, Goldstein, & Brooks, 2014) to provide the healthcare system and the medical staff with information for appropriate intervention consistent with the patients’ needs (Rabe et al., 2007).

ADLs include activities and tasks that people routinely perform in their daily life inside/outside their homes (Barlow, 2012). Vriendt et al. divided ADL into 3 domains namely basic activities of daily living (BADLs) including self-care behaviors, such as dressing and bathing, instrumental activities of daily living (IADLs), such as cooking, house chores, and shopping and AADLs, including voluntary behaviors influenced by cultural and motivational factors, which indicate satisfying activities beyond personal independence. The combination of all three domains of ADL includes all the activities that a person performs in daily life (De Vriendt et al., 2012).

In the past decades, physical activity assessment instruments were traditionally and predominantly used in epidemiological research to measure activity as treatment outcome and an indicator of health (Lagerros & Lagiou, 2007); but new research showed that instruments made for measuring ADL can be used for evaluation of treatment outcomes and planning rehabilitation and care interventions (Palange et al., 2007). Several studies showed that measuring activity by paper-and pencil instruments could evaluate and detect small differences in levels of physical activity created as a result of treatment or a specific intervention. Use of this instrument has become commonplace in research and in clinical practice (Frei et al., 2011). Paper-and-pencil instruments are questionnaires routinely used in clinical practice and in clinical research. These instruments extract self-reported data from patients directly. They are affordable and convenient, do not require special equipment or training, and can easily be performed at any time or place. These instruments and tests can be performed in research and in daily clinical work (Stull, Kline Leidy, Jones, & Ståhl, 2007). There are theoretical arguments regarding the need for an instrument to demonstrate good reliability, validity, and responsiveness (Mokkink et al., 2010a).

A review of studies showed that experts and researchers have used several instruments for measuring ADL in patients with COPD. Despite the importance of ADL measurement, and international guidelines on COPD and its treatment, none suggested a method, an instrument, checklist or a questionnaire to evaluate ADL. Thus, the goal of this study was to review the existing instruments for evaluation of ADL in patients with COPD, and assess psychometrics of instruments according to the COSMIN taxonomy (consensus based standards for the selection of health measurement instrument).

## 2. Materials and Methods

This systematic review was carried out according to the “University of York's Center for Reviews and Dissemination Guidance” (*Systematic Reviews. CRD guidance for undertaking reviews in health care*, January 2009). According to this guideline, the first step is the development of a protocol, which consists of the main goal and a set of predetermined stages and methods to perform a systematic review (Liberati et al., 2009). In this study, the protocol consisted of designing the question for systematic review, inclusion criteria, search strategy, selection and extraction of data, evaluation of quality of studies, data synthesis, and publication of findings determined by the researcher.

As GOLD report states that increasing physical activities is an important goal of treatment in patients with COPD (Pitta et al., 2005), the question addressed in this systematic review was “what are the existing instruments for evaluation of ADL in patients with COPD to assess the efficacy of an intervention for physical activity enhancement”.

### 2.1 Study Selection

Published articles over the past three decades (from 1980 to 2013) in English on ADL in patients with COPD were searched in Web of Science, MEDLINE, Google Scholar, Cochrane, PubMed, ProQuest, and CINAHL data bases using the following keywords: “Chronic obstructive pulmonary disease”, “COPD”, “activity of daily living”, “ADL”, “activities of daily living”, “day to day activities”, “daily life activities”, “daily activities”, “instrument”, “questionnaire”, “test”, “assessment”, “paper-and-pencil instruments”, and “measure”. Following the Internet search, manual search was carried out to find article references relevant to our study. Titles of relevant references were searched and full texts of articles meeting the inclusion criteria were studied.

### 2.2 The Inclusion Criteria for Articles Were

1. Articles written in English.
2. Original articles describing a paper-and-pencil instrument development and validation or psychometric process to evaluate BADL, IADL, and AADL in patients with COPD.

### 2.3 The Exclusion Criteria for Articles Were

3. Any article that included instruments that examined ADL in only one limb.
4. Any article that included instruments that measured ADL in other pulmonary diseases.
5. Any article that included laboratory, semi-laboratory, or field instruments.
6. Any article that used generic instruments to measure ADL in patients with COPD.
7. Articles that used paper-and-pencil instruments to evaluate BADL, IADL, and AADL in patients with COPD.

### 2.4 Quality Appraisal and Data Extraction

The quality of articles that met the inclusion criteria was assessed. The COSMIN checklist for assessing the methodological quality of studies on measurement was used (Mokkink et al., 2010a). The psychometric qualities of each study were independently assessed by two researchers. Disagreements between the researchers were resolved by discussion or by a third researcher. To extract main data from studies, an initial data extraction form was prepared (Table 1). The form was developed by researchers based on the COSMIN taxonomy (consensus based standards for the selection of health measurement instrument) (Mokkink et al., 2010b). This form consisted of questions about specific psychometric criteria. After entering data from four studies, the form was revised, and data from studies were entered in the final form. The most important psychometrics properties according to the COSMIN taxonomy included content validity, construct validity, criterion validity, stability, internal consistency, responsiveness, and interpretability.

**Table 1:** Data extraction form

	Data extraction form
1	Is the tool based on a theoretical framework or a qualitative study?
2	Have patients’ experiences been used in construction of items?
3	Is the tool one dimensional or multidimensional?
4	Have content, construct, and criterion validities been provided?
5	Has reliability of the tool been determined?
6	Has sensitivity of the tool been determined?
7	Has the tool designer identified intended population?
8	Has the tool been designed for a particular group of patients with COPD?
9	Can the tool be used for all patients with COPD (illiterate, low literate, disabled)?
10	Is it easy and simple to use the tool?
11	Is the tool time consuming?
12	Can the tool be used in daily clinical work with the least facilities and equipment?
13	Has the tool been translated into other languages? if not, can it be easily translated?
14	Are there any evidence and documents that the tool has been used in clinic?
15	Are there any guidelines that recommend use of the tool in clinic?
16	Is scoring method simple in this tool?
17	Has the tool designer determined a cut-off point?

## 3. Results

PubMed database search using the above-mentioned keywords yielded 1,463 articles, and following search in other databases, 376 articles were found. After exclusion of repeated articles, titles and abstracts of 1,424 articles were reviewed. At this stage, 1,265 articles were excluded. Ultimately, 159 articles were included in the study for full text review. Then, relevant references, whose full texts had been studied, were manually searched in various databases. Twenty-seven articles were added to the study after manual assessment of references, and the full texts of 186 articles were reviewed. After this evaluation, 155 articles were excluded.

The most common reason for excluding these studies was that they were either laboratory, semi-laboratory, field tests or performance-based tests, compared two instruments, examined daily physical activity in only one limb, used generic instruments to measure ADL, investigated correlation of physical activity with other variables, investigated the effect of COPD on ADL, assessed physical activity in patients with COPD after lung transplant, or assessed instrument for diseases other than COPD.

The remaining 31 articles were evaluated for extraction of data and led to identification of 13 paper-and pencil instruments for investigating ADL in COPD patients.

Flowchart 1 shows article search and selection method in every stage. Of the 13 identified instruments, four instruments (CDLM, MRADL, FPI, FPI-SF) were developed to examine ADL in COPD patients (Table 2 shows instruments that measure ADL in COPD patients).

**Table 2:** Tools that measure activity of daily living in COPD patients

Test	Assessed construct	Scaling	Domains	Number of items	Validity			Reliability		Interpretability	Responsiveness
					V1	V2	V3	R1	R2		
Capacity of daily living during the morning (CDLM) (Partridge, et al., 2010)	BADL	Scoring according to 3- or 4-point Likert scale, depending on type of question (for instance: Yes, I can do this on my own- Yes, but I need help with that- No I cannot do that- for other reasons I cannot do that.	All basic activities that a person performs in the morning, including taking a shower, toweling the body, dressing, preparing breakfast, taking a walk around the house, etc.	6 items	_	_	✓	✓	✓	✓	_
Manchester respiratory ADL questionnaire (MRADL) (Yohannes, Roomi, Winn, & Connolly, 2000)	BADL IADL Leisure activity	4-point Likert scale (not at all, with help, alone but with difficulty, alone with ease)	Basic daily activities that a person performs, and leisure time activities	21 items	✓	_	✓	✓	✓	_	✓
Functional performance inventory (FPI) (Knebel, 2010; Larson, Kapella, Wirtz, Covey, & Berry, 1998; Leidy, 1999; Ozkan, Gemicioglu, Durna, & Demir, 2009)	BADL IADL Leisure activity	4-point Likert scale (from I do this alone, to I cannot do this anymore)	Basic and instrumental daily activities a person performs, and also religious and social activities	65 items	✓	_	✓	✓	✓	_	_
Functional performance inventory-short form (FPI-SF) (Ai-Min Guo, et al., 2011; Leidy, Hamilton, & Becker, 2012)	BADL IADL Leisure activity	4-point Likert scale (from I do this alone, to I cannot do this anymore)	Basic and instrumental daily activities a person performs, and also religious and social activities	32 items	✓	✓	✓	✓	✓	_	_

V1 - Content Validity; V2 - Criterion Validity; V3 - Construct Validity; R1 – Stability; R2 - Internal Consistency.

Nine instruments (LCADL, CARS, ADL-D, PFSS, PFSDQ, PFSDQ-M, PFSS-11, DIRECT, SOBDA) were designed to measure dyspnea as an indicator of ADL (Table 3 shows instruments designed to measure dyspnea as an ADL indicator). FPI and FPI-SF were based on a theoretical framework that accounts for important and effective factors in performing ADL in patients with COPD.

**Table 3:** Instruments were designed to measure dyspnea as an indicator of ADL

Test	Construct	Scaling	Domains	Item number	Validity			Reliability		Interpretability	Responsiveness
					V1	V2	V3	R1	R2		
The London chest ADL scale (LCADL)(Bisca, Proenca, Salomao, Hernandes, & Pitta, 2014; Carpes, Mayer, Simon, Jardim, & Garrod, 2008; Garrido, et al., 2006; R Garrod, Bestall, Paul, Wedzicha, & Jones, 2000; Kovelis, et al., 2011)	Dyspnea	5-point Likert scale (from performing tasks without dyspnea to inability to perform tasks due to dyspnea)	Basic daily life activities and leisure time	15 items	_	✓	✓	✓	✓	_	✓
COPD activity rating scale (CARS)(Morimoto, Takai, Nakajima, & Kagawa, 2003)	Dyspnea and the amount of help the individual needs to perform his daily activities.	3- point Likert scale (dependent, somewhat dependent, and independent)	Basic daily life activities and social activities	12 items	_	_	✓	✓	✓	_	✓
The Nagasaki University respiratory ADL questionnaire (ADL-D)(Yoza, Ariyoshi, Honda, Taniguchi, & Senjyu, 2009)	Dyspnea	5- point Likert scale (from performing tasks without dyspnea to inability to perform tasks due to dyspnea)	Basic activities	15 items	_	✓	✓	_	✓	_	_
Pulmonary functional status scale (PFSS)(Weaver, Narsavage, & Guilfoyle, 1998)	Dyspnea BADL IADL	4- point Likert scale (from performing tasks with huge difficulty to performing tasks without difficulty, and 5- point Likert scale (from not doing the task to doing the task 3 times or more per week)	Basic daily life activities and spiritual, psychological and sexual activities	56 items	✓	✓	✓	✓	✓	_	_
The pulmonary functional status and dyspnea questionnaire (PFSDQ)(Lareau, 1994; Lareau, Carrieri-Kohlman, Janson-Bjerklie, & Roos, 1994)	Dyspnea BADL IADL	Likert scales: 0-7 for activities, 0-10 for dyspnea	Basic daily life activities and social activities and leisure time	164 items	✓	_	✓	✓	✓	_	_
The modified version of the pulmonary functional status and dyspnea questionnaire (PFSDQ-M) (A. M. Guo, Han, Wang, Lin, & Wu, 2010; Kovelis, et al., 2008; Kovelis, et al., 2011; Lareau, Meek, & Roos, 1998; Wingårdh, Engström, & Claesson, 2007)	BADL	Tool has two parts. First it measures patient's dyspnea, and then, daily activities. Scoring is based on 11-point Likert scale (from fully active to fully inactive)	Basic activities that a person performs daily, including: taking a shower, dressing, preparing food, walking	40 items	_	_	✓	✓	✓	_	✓
Short-form pulmonary functional status scale (PFSS-11)(Chen, Narsavage, Culp, & Weaver, 2010; Narsavage, Chen, Culp, & Weaver, 2009)	Dyspnea BADL IADL	4- point Likert scale (from performing tasks with huge difficulty to performing tasks without difficulty, and 5- point Likert scale (from not doing the task to doing the task 3 times or more per week)	Basic daily life activities and emotional activities	11 items	_	_	✓	✓	✓	_	✓
Disability related to COPD tool (DIRECT)(Aguilaniu, et al., 2011)	The amount of inability in performing BADL IADL AADL	Different Likert scales scoring for each question (3, 4, or 5 points)	Basic and advanced daily activities	10 items	✓	✓	✓	✓	✓	_	_
Shortness of breath with daily activities (SOBDA)(W. Chen, et al., 2010; Howard, et al., 2012; Watkins, et al., 2013; T. Wilcox, et al., 2010; T. K. Wilcox, et al., 2013)	Dyspnea in performing BADL IADL	Not explained	Basic and instrumental activities	37 items	✓	✓	✓	✓	✓	_	✓

V1 - Content Validity; V2 - Criterion Validity; V3 - Construct Validity; R1 – Stability; R2 - Internal Consistency.

Two instruments (PFSDQ-M) and (CDLM) were designed to measure BADL, and ADL-D included only dyspnea. Most of them (n=8) combined BADL with IADL and leisure activity. The initial search of the literature revealed that researchers have classified ADL in various forms. But the classification system suggested by Vriendt (De Vriendt et al., 2012) and colleagues has been used in the majority of studies. In fact the majority of instruments only dealt with the two main dimensions of ADLs (BADL and IADL), and did not consider its advanced dimension (AADL), which is influenced by cultural and motivational factors. Just one instrument (CARS) included questions regarding the amount of help an individual needed to perform daily activities. Two instruments namely DIRECT and PFSS addressed sexual activity, and one (FPI) assessed religious activity.

The results showed that construction of ADL measuring instruments varied between studies, but because some of the instruments did not include information about their psychometric properties we could not compare them. In some instruments, patient information had not been used when creating the items (for example “COPD Activity Rating Scale” or CARS). In fact, many instruments followed an unclear process in construction of items, even though using information from individuals for whom the instrument is constructed and should be completed is important. Tables 2 and 3 show the evidence for the psychometric properties of the ADL instruments developed for COPD patients. Validity and reliability were the most common psychometric properties evaluated. Information on content validity of seven instruments (MRADL, FPI-SF, FPI, DIRECT, SOBDA, PFSS and PFSDQ) was available. For these instruments, interviews, focus groups and review of the literature had been performed to provide content validity. Criterion validity was reported for five instruments (FPI-SF, LCADL, ADL-D, PFSS and DIRECT). Information on construct validity was given related to all instruments. All instruments had undergone an evaluation method (stability or internal consistency) for reliability. Information on interpretability was provided only for one instrument (CDLM). Although responsiveness represents an important dimension of psychometric instruments, it was reported for only six instruments (MRADL, LCADL, CARS, PFSDQ-M, PFSS-11 and SOBDA).

## 4. Discussion

In the past decades, physical activity assessment instruments were traditionally and predominantly used in epidemiological research to measure activity as the treatment outcome and an indicator of health (Lagerros & Lagiou, 2007). When the results of several studies showed that activity measuring paper-and pencil instruments can evaluate and detect small differences in level of physical activity created as a result of treatment or a specific intervention, use of this instrument became commonplace in research and in clinical practice (Frei et al., 2011). The increased number of published articles focusing on ADL in patients with COPD indicates that not only lung function, but also activity is impaired in COPD patients (Kocks, Asijee, Tsiligianni, Kerstjens, & van der Molen, 2011). The results showed that only 31 studies assessed psychometric properties of ADL instruments in patients with COPD, and our extensive search strategy led to the identification of 13 paper-and-pencil instruments.

Our study results showed that, although specific instruments exist for measuring ADL in patients with COPD, some experts still measure ADL in patients using generic scales, such as the Barthel index (Lee, Lee, & MacKenzie, 2006); even though inability to perform ADL in patients with COPD is different from other diseases and conditions. This difference originates from the fact that due to dyspnea COPD patients are sometimes unable to carry out the task, despite having the physical capacity to do it (Yohannes, Baldwin, & Connolly, 2002).

Despite the importance of ADL measurement in COPD patients, and large number of professional scientific and international guidelines on COPD and how to treat and care for it, none of these guidelines have suggested an appropriate instrument, or checklist for measuring ADL (Pauwels, Buist, Calverley, Jenkins, & Hurd, 2012). Janaudis believes that lack of attention to ADL measurement in COPD patients is due to the clinical staff's lack of time to test instruments, or use a method beyond assessment of lung function and oxygenation, which is frequently used today or lack of knowledge about the most appropriate ADL instrument or questionnaire for measuring ADL in this particular patient group (Janaudis-Ferreira et al., 2014).

Although the American Association of Psychology (APA) has clearly stated that “the construct that the instrument is to measure must be expressed in a theoretical framework” (“American Educational Research Association, American Psychological Association, National Council on Measurement in Education: Joint Committee on Standards for Educational Psychological Testing. Standards for educational and psychological testing, 1999), our study shows that none of the existing instruments is based on a proper theoretical framework that accounts for important and effective factors in performing ADL in patients with COPD. While, in patients with COPD, ADL depends on a number of variables like symptoms of the disease, fitness, level of independence and level of need for mobility aids or other people's help. The majority of instruments do not account for these. Not establishing these instruments on a proper theoretical framework is indicative of inadequate and incorrect perception of the concept of daily physical activity in patients with COPD. One of the problems in this regard is the inability to measure changes in physical activity resulting from treatment or rehabilitation and interventions to improve physical activity in patients with COPD because the instrument cannot accurately measure the concept of daily physical activity.

Physical activity is a complex construct made up of many concepts. The construct of physical activity can include the ability to perform ADL and its associated symptoms, such as pain and dyspnea. As the present study shows, in most ADL measuring instruments, the construct measured is either BADL or IADL, while according to new definitions, ADL consists of three dimensions. Therefore, the majority of instruments do not measure the third dimension of ADL (AADL), which includes “voluntary behaviors affected by cultural and motivational factors, and indicate satisfactory activities beyond personal independence” (De Vriendt et al., 2012). When a study only aims to measure BADL or IADL, using an existing instrument would be suitable, but, if the researcher intends to measure all three dimensions of ADL, they will have serious difficulties in finding an appropriate measure. Considering that one of the aims of systematic review studies is to identify the need for further studies, there is a clear need for the development of an instrument that is derived from the concept of ADL in patients with COPD that evaluates all three dimensions of ADL.

Paper-and-pencil instruments should have strong psychometric features, especially in terms of content validity of their items. Content validity is indicative of how accurately the instrument can measure the construct for which it was designed. Paper-and-pencil instruments should also have high construct validity and reliability (Frei et al., 2011). Furthermore, since they should be able to identify small changes, they should have a high sensitivity to change (Revicki, Hays, Cella, & Sloan, 2008). However, the current study shows that the most important limitation for selection of instruments for measuring ADL in patients with COPD is the lack of information about psychometrics of the instrument. Psychometrics has either not been fully investigated with the instruments or has not been sufficiently reported. For instance, regarding interpretability and sensitivity of instruments, very few studies have mentioned these two dimensions of psychometrics. In most studies, attention had been paid to construct validity, and to a lesser extent to content validity. Only half of the studies used in the present investigation had gone through a thorough content validation process, and had used an empirical, qualitative approach, such as an interview, for constructing items. The lack of full psychometric information limits the evidence-based selection of a suitable instrument for researchers and clinicians.

Study results showed that only one of the ADL instruments was designed according to cultural and lifestyle dimensions and other instruments had not considered this issue; while, clearly, people's culture and lifestyle vary in different countries, and as a result their ADL will also be different. However, the AADL dimension is affected by culture and lifestyle more than the other two dimensions.

None of the instruments specified the group of patients the instrument was developed for. A strong point in paper-and-pencil instruments is that they can be used in both hospital and laboratory settings and in the patients’ real life environment. Most paper-and-pencil instruments are designed in such a way that illiterate, low literate, disabled, and frail people cannot answer them. People with mobility or comprehension problems such as patients with reading and comprehension problems cannot answer these questions either. When these people are not accounted for in designing of instruments, a proportion of the population with COPD and their right for evaluation will be neglected (Jahagirdar, Kroll, Ritchie, & Wyke, 2013).

The ultimate aim of all studies conducted on clinical instruments or tests should be the assessment of ADL effects on management and treatment outcomes of patients (Craig et al., 2011). Systematic review of studies conducted for construction of clinical instruments or tests should draw researchers’ attention toward weaknesses and strengths of studies in order to propose the need for better studies on intended instruments (Henry & Hayes, 2006). However, no systematic review can provide a definite estimate of accuracy and rigor of a test or an instrument; it can only reduce the gap between correct clinical decisions and evidence, and provide a basis for future studies (*Systematic Reviews. CRD guidance for undertaking reviews in health care*, January 2009).

In conclusion, the current study revealed that psychometric properties were not complete for any of the tools, which measure ADL in patients with COPD. We recommend that a proper conceptual framework first be designed for daily physical activity in patients with COPD, followed by a paper-and-pencil instrument for measuring daily physical activity in these patients. Despite several ADL instruments identified, complete psychometric process has been done in only a few. Selection of the appropriate instrument should focus on the aim of the study and the target construct. The strength of this study was in researchers’ adherence to methodology of systematic reviews in studies that expressed how systematic reviews should be performed. Although researchers tried to adhere to the protocol in all stages of the study and gather all relevant articles in the first stage of the study, there is still a chance that a relevant tool may have been missed. Sometimes, there is a difference of opinion about the inclusion or exclusion of a tool, which takes a long time to agree upon. In such cases, researchers tried to consider the most scientifically defensible criterion in changing the protocol, and to remain faithful to the protocol in all stages of the study.

The most important limitation of this systematic review was that many tools used different surrogate or substitute terms, such as the phrase “functional status”, and argued that functional status indicated level of participation of the person in activity, and that it was synonymous to “activities of daily living” [108]. However, considering that such synonymous or substitute statements were not part of the study inclusion criteria, these tools were excluded. It is recommended that a study be conducted to analyze the concept of ADL in patients with COPD, to clarify if ADL construct is synonymous or a surrogate of statements or constructs.
